# The crosstalk between anoikis and epithelial-mesenchymal transition and their synergistic roles in predicting prognosis in colon adenocarcinoma

**DOI:** 10.3389/fonc.2023.1184215

**Published:** 2023-06-07

**Authors:** Jiahui Zhou, Sheng Yang, Dawei Zhu, Hao Li, Xinsheng Miao, Menghui Gu, Wei Xu, Yan Zhang, Wei Tang, Renbin Shen, Jianhua Zha, Jianhua Zhu, Zheng Yuan, Xinhua Gu

**Affiliations:** ^1^ Department of Gastrointestinal Surgery, The Affiliated Suzhou Hospital of Nanjing Medical University, Suzhou Municipal Hospital, Gusu School, Nanjing Medical University, Suzhou, China; ^2^ Department of Colorectal Surgery, The First Affiliated Hospital of Nanjing Medical University, Nanjing, China; ^3^ Colorectal Institute of Nanjing Medical University, Nanjing, China

**Keywords:** colon adenocarcinoma, anoikis, EMT, crosstalk, risk model

## Abstract

Anoikis and epithelial-mesenchymal transition (EMT) are significant phenomena occurring in distant metastasis of colon adenocarcinoma (COAD). A comprehensive understanding of their crosstalk and the identification of key genes are vital for treating the distant metastasis of COAD. The objective of this study was to design and validate accurate prognostic predictors for COAD patients based on the anoikis and EMT processes. We obtained gene signatures from various databases and performed univariate and multivariate Cox regression analyses, principal component analysis (PCA). The COAD patients were categorized into the worst prognosis group, the Anoikis Potential Index (API) Low + EMT Potential Index (EPI) High group and the others group. Then we utilized gene set enrichment analysis (GSEA) to identify differentially expressed genes and to establish a prognostic risk model. The model classified patients into high- or low-risk groups, with patients in the high-risk group displaying worse survival status. A nomogram was established to predict overall survival rates, demonstrating high specificity and sensitivity. Additionally, we connected the risk model to the tumor microenvironment (TME) using single-sample GSEA and the MCP counter tool, as well as evaluated the sensitivity to common chemotherapeutic drugs, such as Gefitinib and Gemcitabine. Lastly, cell and tissue experiments suggested a positive correlation among anoikis resistance, EMT, and liver/lung metastasis of COAD. This is the first study to comprehensively analyze the crosstalk between anoikis and EMT and offers new therapeutic targets for COAD metastasis patients.

## Introduction

According to the statistics presented by the American Cancer Society (2023), COAD ranks third in terms of the incidence and mortality rate of all cancers, irrespective of gender. It affects young individuals and poses a serious health risk to the public (1). It has been reported that Stage I patients can attain a 5-year survival rate (after surgical resection) of ≥90%, while the patients with distant metastasis showed a 5-year survival rate of only 11%, despite the application of adjuvant chemotherapy, targeted drugs, or immunotherapy (2, 3). This has necessitated the need to thoroughly understand and urgently address the problem of COAD metastasis.

EMT is a phenomenon where epithelial cells can acquire a mesenchymal phenotype, which is first observed in embryonic development. Once EMT is activated, tumor cells undergo many changes, such as their dissociation with tight junctions, disruption of apical-basal polarity, and remodeling of cytoskeletal structures, all of which contribute to the movement of cells from their primary location, invasion of neighboring tissues, survival during the circulation process, and the eventual formation of metastatic foci at distant sites (4, 5). Several studies have reported an association between EMT and COAD metastasis. Wang et al. revealed that the THZ1 could promote EMT by inhibiting the degradation of Snail, which in turn increased colorectal cancer liver metastasis (6). Xiang et al. demonstrated that Snail could facilitate the formation of M2-macrophages by secreting CXCL2, which finally promoted the lung metastasis of colorectal cancer (7).

Epithelial cells increase their survival rate by attaching to the ECM. In addition, they undergo apoptosis after they get detached from the ECM, which is defined as the anoikis phenomenon (8). It was noted that the tumor cells acquire resistance to anoikis, where even when they get detached from the ECM they cannot undergo apoptosis easily, invade the surrounding tissues, and subsequently metastasize distantly (9). Many recent studies have highlighted the correlation between anoikis and COAD metastasis. Wei et al. reported that simultaneous inhibition of PDK1 and STAS3-Y705 enhanced the anoikis process, which further inhibited colorectal cancer liver metastasis (10). Xu et al. demonstrated that CPT1A-mediated FAO activation promoted anoikis resistance in colorectal cancer cells and improved lung metastasis (11).

Both the EMT and anoikis phenomena occur during the invasive stage of primary tumors and undergo a few crosstalks (12). It was observed that up-regulated Claudin-1 reduced E-cadherin expression *via* ZEB-1 modulation, attenuating the invasive ability, and anoikis of COAD cells (13). Up-regulated miR-450a was reported to inhibit EMT, promote anoikis, and thus inhibit the migration and invasion capacities of ovarian cancer cells (14). The deletion of 4.1N facilitated EMT, anoikis resistance, and consequently the metastasis of epithelial ovarian cancer cells (15). Therefore, the co-analysis of EMT and anoikis could help to identify the genes that play key roles in COAD metastasis.

**Figure 1:**
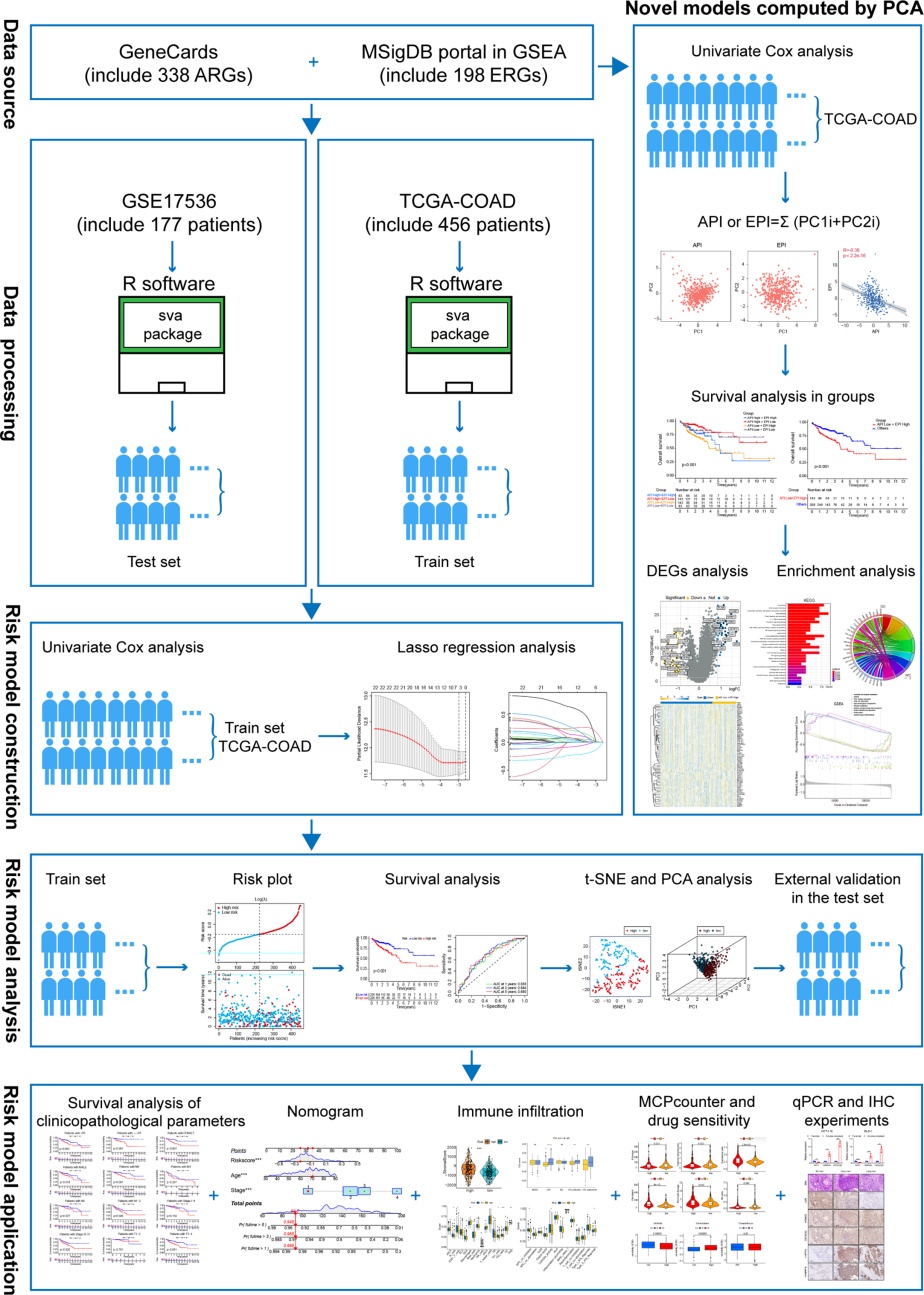
The flowchart of overall study methods and results.

This study comprehensively and substantively described the interaction between anoikis and EMT. We divided COAD patients into the worst prognosis group and the others groups by univariate and multivariate Cox regression analysis and PCA. We then used GSEA to identify differentially expressed genes and to establish a prognostic risk model containing NAT1, CDKN2A, and PCOLCE2 with high specificity and sensitivity. In addition, we linked the risk model to the TME and assessed the sensitivity to common chemotherapeutic drugs. Finally, cell and tissue experiments further demonstrated the correlation between anokis, EMT and COAD metastasis. **Figure 1** depicts the flowchart used in this study.

## Materials and methods

### Data sources and analysis

A total of 338 ARGs (anoikis-linked genes) were obtained from GeneCards (https://www.genecards.org/), and the genes showing a relevance score of >1 were chosen in the study. The EMT signature containing 198 genes (EMT-related genes, i.e., ERGs) was derived from the MSigDB portal in GSEA (http://software.broadinstitute.org/gsea/msigdb). The genetic information and clinically-relevant data from The Cancer Genome Atlas (TCGA) database (https://portal.gdc.cancer.gov/repository) was acquired for 459 COAD patients. Meanwhile, gene expression matrices containing 317 ARGs and 195 ERGs were extracted independently using R language software, and the ‘limma’ software was used to analyze the variations between the two. Thereafter, 152 DEGs for ARGs (diff-ARGs) and 125 DEGs for ERGs (diff-ERGs) were selected based on the following screening criteria: |FC|>1.5 and p<0.05. The ‘Rcircos’ software was applied to map the location of anoikis and EMT-related genes on human chromosomes. The data associated with the somatic mutations, genome mutations, and Copy number variations (CNV) in COAD were also derived from TCGA.

### Survival analysis

In this study, univariate and multivariate Cox regression analyses were carried out with the help of the ‘survival’’ package. The Kaplan Meier (KM) curves were plotted to compare the differences (variations) in overall survival (OS) between different groups. The logarithmic rank test was employed to determine the P-value between different groups. Furthermore, the time-dependent receiver operating characteristic (ROC) curves were assessed using the ‘survivalROC’ packets. The area under the ROC curve (AUC) value was used to evaluate the prognostic performance of the ROC curve.

### Computational models of anoikis and EMT levels in COAD

In this study, Principal component analysis (PCA) was conducted for determining the anoikis and EMT scores for understanding the degree of anoikis and EMT in each sample. Then, univariate Cox regression analysis was conducted for analyzing the survival of anoikis and EMT-related genes. Then, the gene expression matrix (P<0.05) for PCA was extracted, and the principal components 1 and 2 were determined as the main subjects. Based on a few earlier reports (16–18), the Anoikis Potential Index (API) and EMT Potential Index (EPI) were defined, respectively: API or EPI=Σ (PC1i+PC2i), where *i* denotes the expression of related genes.

### Gene set enrichment analysis

GSEA was utilized to study the relationship between both groups after dividing the TCGA samples into the API Low + EPI High group and the others group. For each analysis, 1000 genome permutations were carried out. The enrichment function was selected using the below-mentioned criteria: the ‘clusterProfiler’ software was used to enrich and evaluate the gene set with the false discovery rate (FDR) of 0.25 and 0.05 NOM p-value. The first five up- and down-regulated functions of the two enrichment sets were visualized by multiple GSEA maps. Then, the DEGs were identified using the differential expression analysis. Thereafter, the ‘ClusterProfiler’ was employed for carrying out Gene ontology (GO) and Kyoto Encyclopedia of Genes and Genomes (KEGG) enrichment analyses for enriching and analyzing the DEGs. Finally, the ‘Enrichplot’ and ‘ggplot2’ software were used for presenting the enrichment results as a bar graph, bubble graph, chord graph, and cluster circle graph.

### Construction of an anoikis-related and EMT-related prognostic model

In this study, univariate Cox regression analysis was conducted for identifying 12 anoikis-linked genes and 11 EMT-related genes. The ‘glmnet’ software was utilized for determining the optimal value of the penalty parameter, lambda, through 1000 times of cross-validation with the Least absolute shrinkage and selection operator (LASSO) regression technique. The risk scores for every COAD patient were estimated using the coefficient and expression of the candidate prognostic anoikis-related genes and EMT-related genes (PAEG), with the following risk scoring formula: $\sum_{i=1}^{n}C\text{oefi }*\text{ Expri}$ ; where Coefi denotes the coefficient of gene *i*, and Expri denotes the expression of every gene in patient *i* (18).

### Validating the PAEG risk model

The expression levels of 3 mRNAs, risk score, survival duration, and risk level for each TCGA sample were integrated into a table and used as a training set. The GSE17536 dataset was used as the test set to validate the training set’s accuracy. The risk prognosis was examined using the KM chart and a log-rank test. Also, the ROC curve was plotted using the ‘timeROC’ tool. Then, the risk heat map, survival state diagram, and risk curve were generated using the ‘pheatmap’ program. PCA and t-SNE analysis were carried out using the ‘Rtsne’ and ‘ggplot2’ packages, respectively. Lastly, the risk score values were combined with clinical parameters (such as age, sex, grade, and TNM stage) and visualized using the forest map.

### Nomogram construction and calibration

In this study, clinical characteristics such as age, TNM stage, and risk scores were used as research subjects for univariate Cox analysis. The survival rates of COAD patients after one, three, and five years were anticipated by a nomograph using the ‘RMS’ tool. Here, calibration curves were used to evaluate the nomogram’s accuracy. Lastly, the ‘ggDCA’ software was used for plotting the decision curve analysis (DCA) curve for predicting the clinical values of various objects.

### Immune infiltration levels in the high- and low-risk groups

Firstly, the ‘ESTIMATE’ software was employed for assessing the stromal score, immune score, and tumor purity between both groups. Then, the differences in immune function, the activity of immune cells, and the immune pathways between the two groups in the training set and test set were examined using the single sample GSEA (ssGSEA) test, and the data were visualized using the box graph. Based on the COAD expression matrix, the ‘MCP counter’ web tool was used for estimating the abundance of various non-immune and immune stromal cells. The data were then observed using violin plots. Then, the human leukocyte antigen (HLA) gene expression and the expression levels of various immune checkpoint genes in both groups were estimated.

### Prediction of chemosensitivity

In this study, the half maximal inhibitory concentration (IC_50_) of three commonly used chemotherapeutic drugs, such as Gefitinib, Gemcitabine, and Camptothecin, was determined in colorectal cancer using the ‘pRRophic’ package. Thereafter, the difference in the sensitivity levels of the above chemotherapy drugs between the high- and low-risk groups was assessed by comparing the variations in the IC_50_ values between both groups.

### Patient tissue specimens and cell lines

Herein, the tissue specimens of COAD patients without distant metastasis, with liver metastasis, and with lung metastasis were extracted from the patients after surgical resection in the Department of Gastrointestinal Surgery, Suzhou Municipal Hospital, Jiangsu, China. The patients had not undergone preoperative chemoradiotherapy, and all specimens were sampled within 10 mins after resection and subsequently fixed in 10% formalin. The Ethics Committee in the hospital approved the experimental procedures used in the study, and the patients were also asked to sign a consent form.

The HCT-116 and DLD-1 colon cancer cell lines were supplied by the Shanghai Cell Bank, Chinese Academy of Sciences (China). These cells were cultured in the DMEM medium (Hyclone, USA) containing 10% (v/v) fetal bovine serum (Hyclone, USA), and 1% (v/v) penicillin/streptomycin solution (Beyotime, China), at 37°C, under 5% CO2 and 95% humidity conditions. The anoikis-resistance model was developed based on published literature (19, 20). Herein, the above-mentioned COAD cell lines were continually cultivated in ultra-low-attachment 6-well cell culture plates (Corning, USA) for 7 days and were transferred to the normal culture plates for 24 h. The re-adhered cells were defined as anoikis-resistant cells. All the experiments were conducted using mycoplasma-free cells. The Shanghai Cell Bank of the Chinese Academy of Sciences (China) validated all the cell lines used in the past three years.

### RNA extraction and qRT-PCR analyses

The TRIzol reagent (Takara, Japan) was used for isolating the total RNA samples from the normal and anoikis-resistant cells. These RNA samples were reverse-transcribed into cDNA using the HiScript II RT SuperMix qPCR kit (Vazyme, China). The qRT-PCR experiments were carried out with the aid of the SYBR Premix Ex Taq Kit (Takara, Japan) on an RT-PCR instrument (7500 Sequence Detection System, Applied Biosystems, USA). The primers were designed and acquired from RiboBio (China). In this study, GAPDH was employed as the internal control for all experiments. The gene expression was represented using the 2^-ΔΔCT^ technique. **Table S1** lists the primer sequences used in the study.

### Hematoxylin-eosin staining and immunohistochemistry

Herein, H&E staining and IHC experiments were conducted as mentioned in an earlier study (21). **Table S2** lists the antibodies used in this study.

### Statistical analysis

All data were statistically analyzed using the R language (ver. 4.1.2) and GraphPad Prism software (ver. 8.0.1). The Kolmogorov-Smirnov normality test was carried out for determining if the data followed the Gaussian distribution, and the data were compared for every sample. If the data conformed to a non-Gaussian distribution, a non-parametric test (Wilcoxon rank test or Spearman correlation) was carried out. On the other hand, when the data conformed to a Gaussian distribution, the parametric test was conducted (unpaired Student’s test, one-way ANOVA, or Pearson correlation). Values with *P<0.05* were deemed statistically significant.

## Results

### Identifying the differentially expressed ARGs and ERGs associated with prognosis

**Figure 2:**
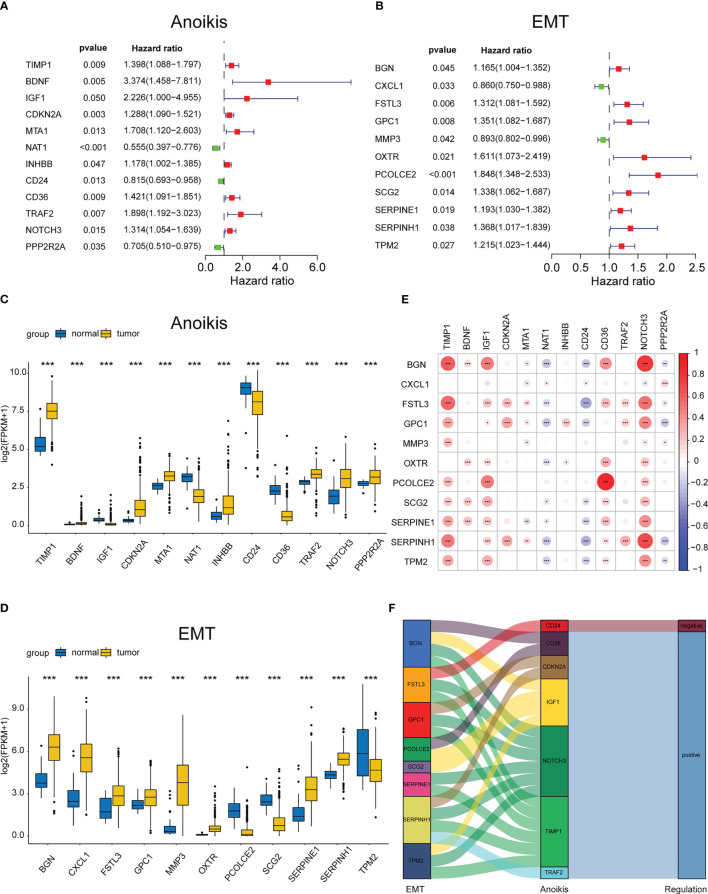
Identifying the differentially expressed ARGs and ERGs associated with prognosis. Forest map of 11 prognostic anoikis-related genes **(A)** and 12 prognostic EMT-related genes **(B)** by univariate Cox analysis (P< 0.05). The differential expression box plot of 11 prognostic anoikis-related genes **(C)** and 12 prognostic EMT-related genes **(D)** in COAD. **(E)**Pearson correlation analysis of 11 prognostic anoikis-related genes and 12 prognostic EMT-related genes. The red color represents a positive correlation; the blue color represents a negative correlation. **(F)** The Sankey diagram displayed the relationship between 11 prognostic anoikis-related genes and 12 prognostic EMT-related genes. *p< 0.05, **p< 0.01, and ***p< 0.001.

Initially, the ARGs and ERGs were downloaded from GeneCards and GSEA, and subsequently, the diff-ARGs and diff-ERGs were selected, respectively. The locations of diff-ARGs and diff-ERGs on human chromosomes were mapped separately in **Supplementary Figure 1A, B**. Then, the clinical prognostic data of COAD patients were acquired from TCGA, and subsequently, 152 diff-ARGs and 125 diff-ERGs were combined with the clinical data using univariate Cox analysis (P<0.05), to eventually obtain 12 prognosis-related ARGs (TIMP1, BDNF, IGF1, CDKN2A, MTA1, NAT1, INHBB, CD24, CD36, TRAF2, NOTCH3, PPP2R2A) and 11 prognosis-related ERGs (BGN, CXCL1, FSTL3, GPC1, MMP3, OXTR, PCOLCE2, SCG2, SERPINE1, SERPINH11, TPM2) (**Figure 2A, B**). Thereafter, the expression levels of the 23 genes in 521 COAD samples (41 normal samples and 480 tumor samples) from TCGA were determined, and box plots were generated independently, which indicated that the genes were differentially expressed between healthy and malignant tissues (**Figures 2C, D**). Meanwhile, the correlations between these 12 ARGs and 11 ERGs associated with prognosis were further analyzed and plotted using the ‘corrplot’ package (**Figure 2E**). Then, the pairs of genes for the Sankey diagram (P<0.05, |cor|>0.3) were selected, where the positive value represented the positive relationship, whereas the negative value indicated a negative correlation (**Figure 2F**).

### Construction of API and EPI with negative correlation

**Figure 3:**
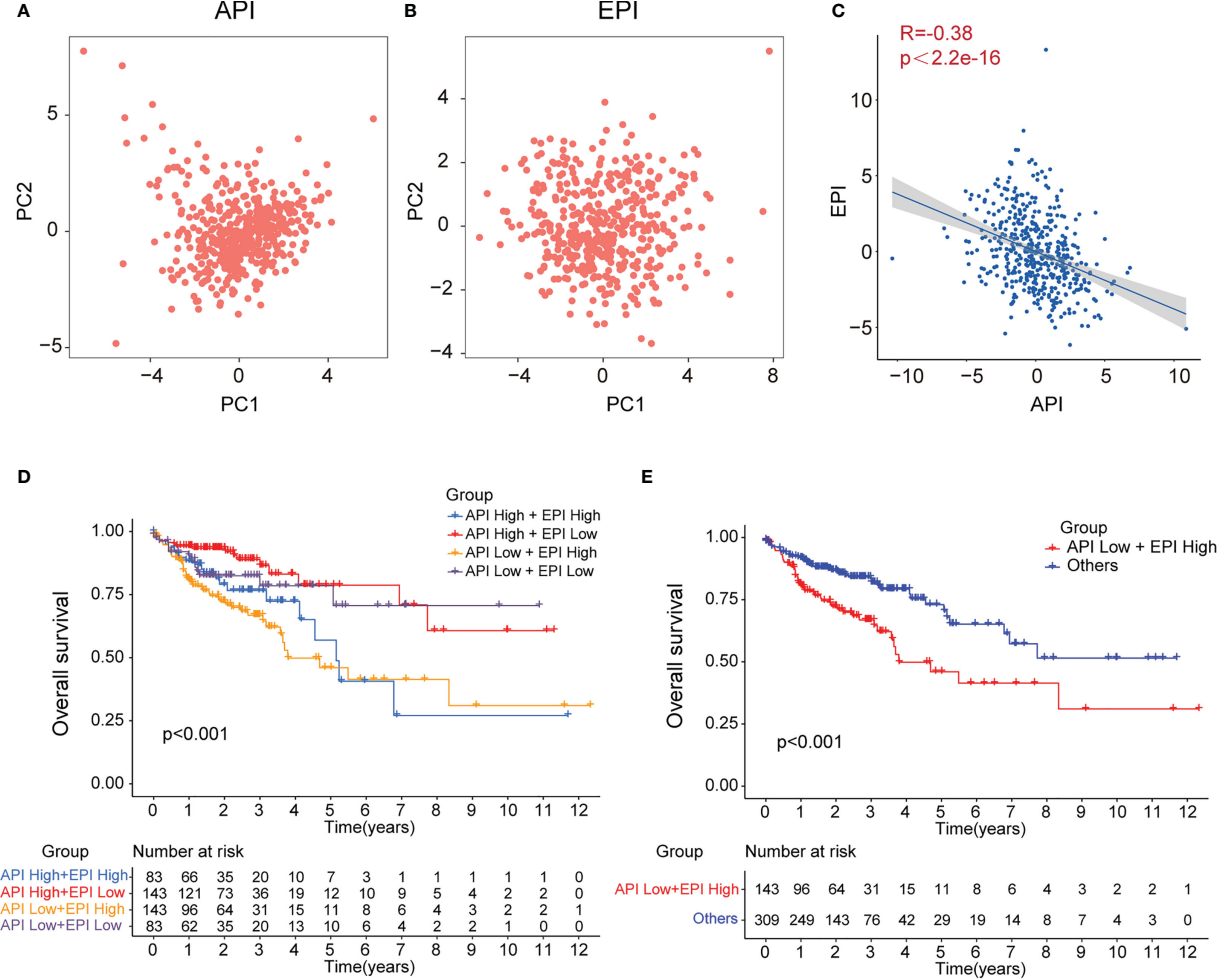
Construction of API and EPI with negative correlation. PCA analysis of 11 prognostic anoikis-related genes **(A)** and 12 prognostic EMT-related genes **(B)**. **(C)**Scatter plot showing the spearman correlation of API and EPI. The KM plot showing overall survival in 4 groups **(D)** and two newly defined groups **(E)**.

To further investigate the crosstalk between ARGs and ERGs, the anoikis and EMT levels in each tumor tissue were calculated and quantified based on the 12 prognosis-related ARGs and 11 prognosis-related ERGs with PCA, and the API and EPI were separately defined (**Figure 3A, B**). A negative correlation was detected between API and EPI in COAD patients (**Figure 3C**). Subsequently, the COAD patients were classified into four molecular subtype groups based on their API and EPI scores, namely API High + EPI High; API High + EPI Low; API Low + EPI High; and API Low + EPI Low. The results of the prognosis analysis indicated that patients in the API Low + EPI High group exhibited the shortest survival time compared to the other three groups (**Figure 3D**). Then, the data from the other groups of COAD patients were combined and compared with the API Low + EPI High group for prognostic analysis, and the corresponding p-value<0.001 was obtained (**Figure 3E**). Both analysis results indicated that high anoikis resistance and high EMT levels were associated with an unfavorable prognosis.

### Enrichment analysis of DEGs in the ‘API Low + EPI high’ and ‘the others’ group

**Figure 4:**
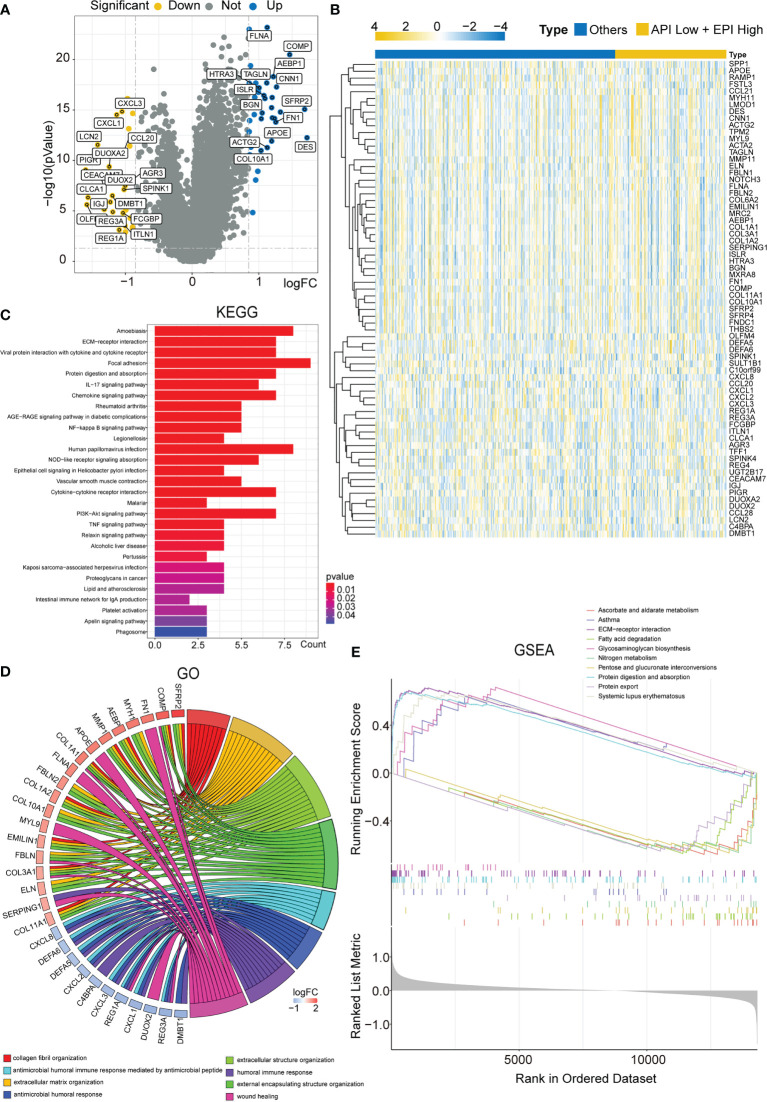
Enrichment analysis of DEGs in the ‘API Low + EPI high’ and ‘others’ group. **(A)**Volcano plots displaying the DEGs between two groups. **(B)** Heatmap created by the DEGs between two groups. The results of KEGG and GO enrichment analysis of the DEGs between two groups showing by barplot **(C)** and chord diagram **(D)**. GSEA results illustrating ten significant enrichments of KEGG in two groups.

Subsequently, the DEGs in the API Low + EPI High group were analyzed and compared to the others groups. Based on the criterion of |FC| > 1.8, 70 DEGs were screened, and a volcano map was plotted (**Figure 4A**) using the clustering heat map (**Figure 4B**). KEGG analysis that was drawn using the barplot indicated that the DEGS in the two groups were primarily enriched in “Focal adhesion”, “Viral protein interaction with cytokine and cytokine receptor”, “ECM-receptor interaction”, “NF-κB signaling pathway,” “IL-17 signaling pathway”, “Chemokine signaling pathway”, “Cytokine-cytokine receptor interaction”, and “PI3K-Akt signaling pathway” (**Figure 4C**). GO analysis plotted by chord diagram suggested a major difference in molecular functions (MF), biological process (BP), or cellular components (CC) in both groups. The DEGs were mainly enriched in “collagen fibril organization”, “extracellular matrix organization”, “extracellular structure organization”, “antimicrobial peptide-mediated antimicrobial humoral immune response”, “external encapsulating structure organization”, “humoral immune response”, “antimicrobial humoral response”, and “wound healing” (**Figure 4D**). The GSEA enrichment analysis between the two groups was described using multiple GSEA diagrams based on the following filtering criteria: FDR<0.25 and NOM P<0.05 (**Figure 4E**). The top five functions that were enriched in the API Low+EPI High group were “Asthma”, “ECM-receptor interaction”, “Glycosaminoglycan biosynthesis”, “Protein digestion and absorption” and “Systemic lupus erythematosus”. The five leading functions enriched in the others group were recorded to be “Ascorbate and alternate metabolism”, “Fatty acid degradation”, “Nitrogen metabolism”, “Pentose and glucuronate interconversions,” and “Protein export”.

### Construction of the prognostic ARGs and ERGs (PAEG) risk model, PCA analysis, and survival analysis of clinicopathological parameters

**Figure 5:**
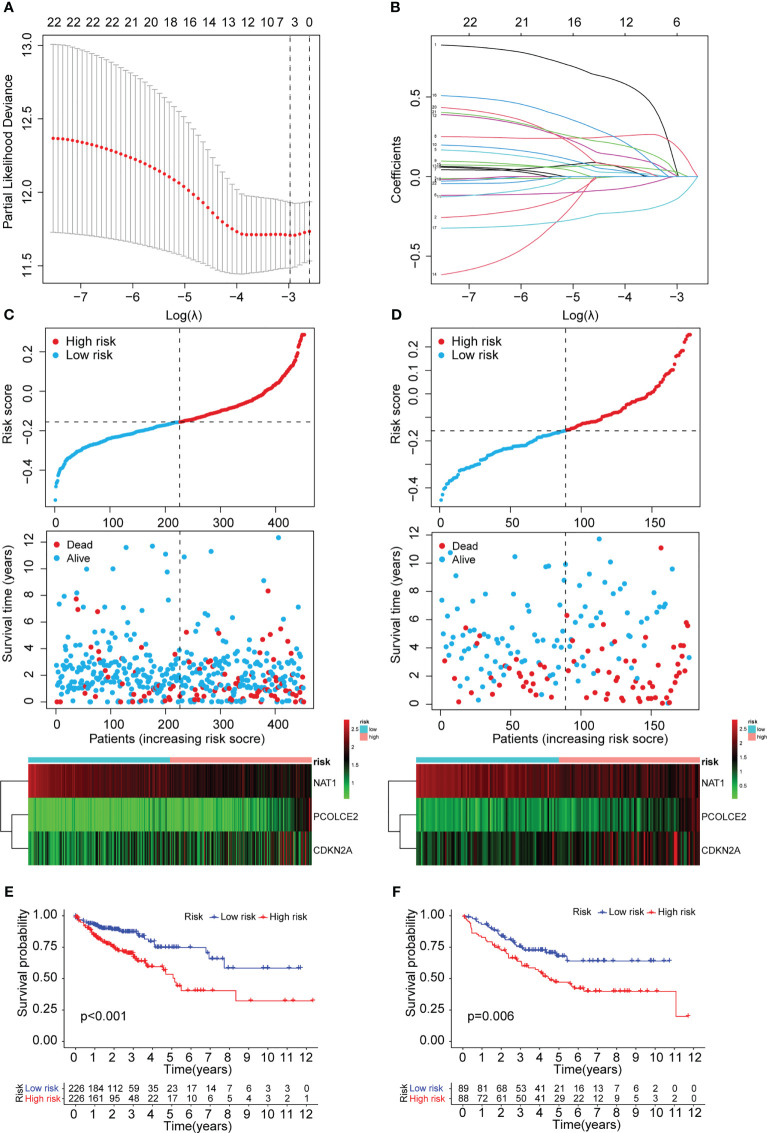
Construction of the prognostic ARGs and ERGs (PAEG) risk model. **(A)** 1000 cross-validation to determine the optimal penalty parameters lambda. **(B)**Lasso regression of the 11 prognostic anoikis-related genes and 12 prognostic EMT-related genes. Scatter plot showing risk score distribution of high-risk and low-risk and the relationship between survival time and risk score based on the training set **(C)** and test set **(D)**. KM plot showing overall survival in training set **(E)** and test set **(F)**.

**Figure 6:**
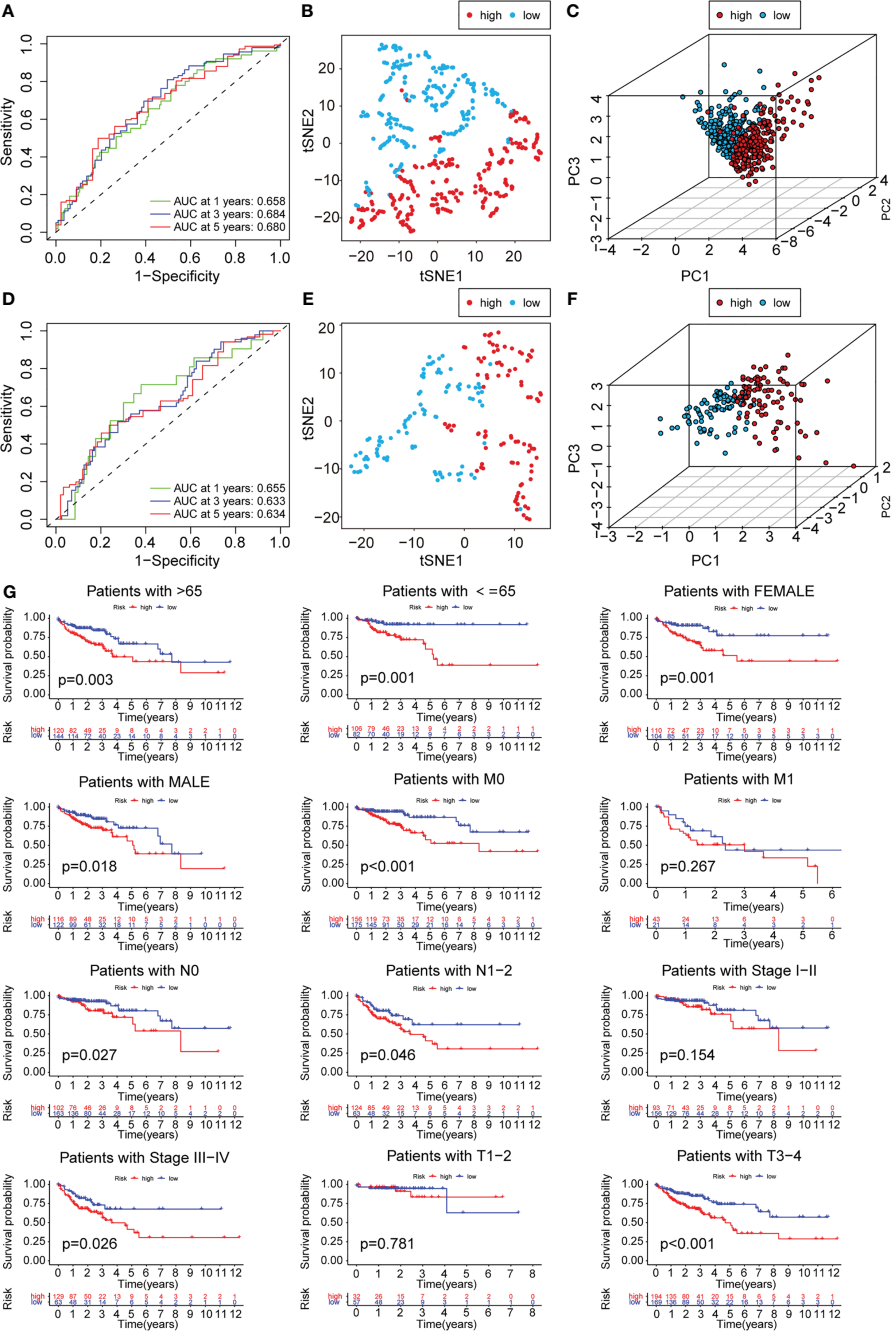
PCA analysis and survival analysis of clinicopathological parameters. The 1-,3- and 5-year ROC curve of risk score in training set **(A)** and test set **(D)**. The t-SNE analysis of training set **(B)**and test set **(E)**. The 3D scatter plot of PCA results of training set **(C)** and test set **(F)**. **(G)** KM plot in subgroups including gender, age and tumor stages.

To construct a risk model for COAD patients based on anoikis and EMT, the 12 prognosis-associated ARGs and 11 prognosis-associated ERGs were combined, and the 23 genes were then subjected to Lasso regression analysis (**Figure 5A, B**). The corresponding coefficient criterion was evaluated by 1,000-fold cross-validation. the optimal penalty parameter lambda was determined, and the corresponding coefficient criterion was calculated depending on a minimum criterion. Lastly, a three-mRNA (NAT1, PCOLCE2, CDKN2A) prognostic risk model was constructed. The risk score was calculated using the following formula: risk score = (-0.135312062940216 ×NAT1 expression) + (0.178733977096469 × PCOLCE2 expression) + (0.0267778987311829 ×CDKN2A expression). The COAD patients were classified into high-risk or low-risk groups depending on risk score values. Then, the GSE17536 data set was selected as the test set for verification, whereas the TCGA data set (n = 452) was chosen as a training set. The risk curve, scatter plot, and risk heat maps were used for highlighting the relationship between the survival time, risk score, and abundance of three genes in COAD patients, determined using the training (**Figure 5C**) and test sets (**Figure 5D**). The results of the prognostic analysis revealed that high-risk patients exhibited a short survival duration compared to the low-risk patients in the training (p<0.001) (**Figure 5E**) and test sets (p=0.006) (**Figure 5F**). Furthermore, ROC curves were used for characterizing the specificity and sensitivity of the risk model. For the 1-, 3-, and 5-year risk scores, the area under the ROC curve (AUC) values in the training set were 0.658, 0.684, and 0.680 (**Figure 6A**), respectively, whereas the test set’s corresponding values were 0.655, 0.633, and 0.634 (**Figure 6D**). Also, the Rtsne package and ggplot2 packages were employed for plotting the t-SNE analysis images from the training set (**Figure 6B**) and test set (**Figure 6E**), independently. The scatterplot3d program was utilized to capture the 3D images of the PCA analysis of the training (**Figure 6C**) and test sets (**Figure 6F**). The above findings highlighted the fact that the high- and low-risk patients showed a favorable heterogeneity in the training and test sets. Finally, the COAD patients were sub-classified in the training set depending on their clinical traits (such as gender, Stage, age, and TNM stage), and the survival durations of high- and low-risk patients were compared after subclassification. The KM curves revealed that a few of the subgroups such as age (both ≤ 65 and >65), gender (male and female), M0 (patients with no distant metastasis), N0 or N 1-2 (patients with or without lymph nodes metastasis), stage III-IV, and T 3-4 (**Figure 6G**) showed a significant survival duration (P< 0.05).

### Cox regression analysis and nomogram development

**Figure 7:**
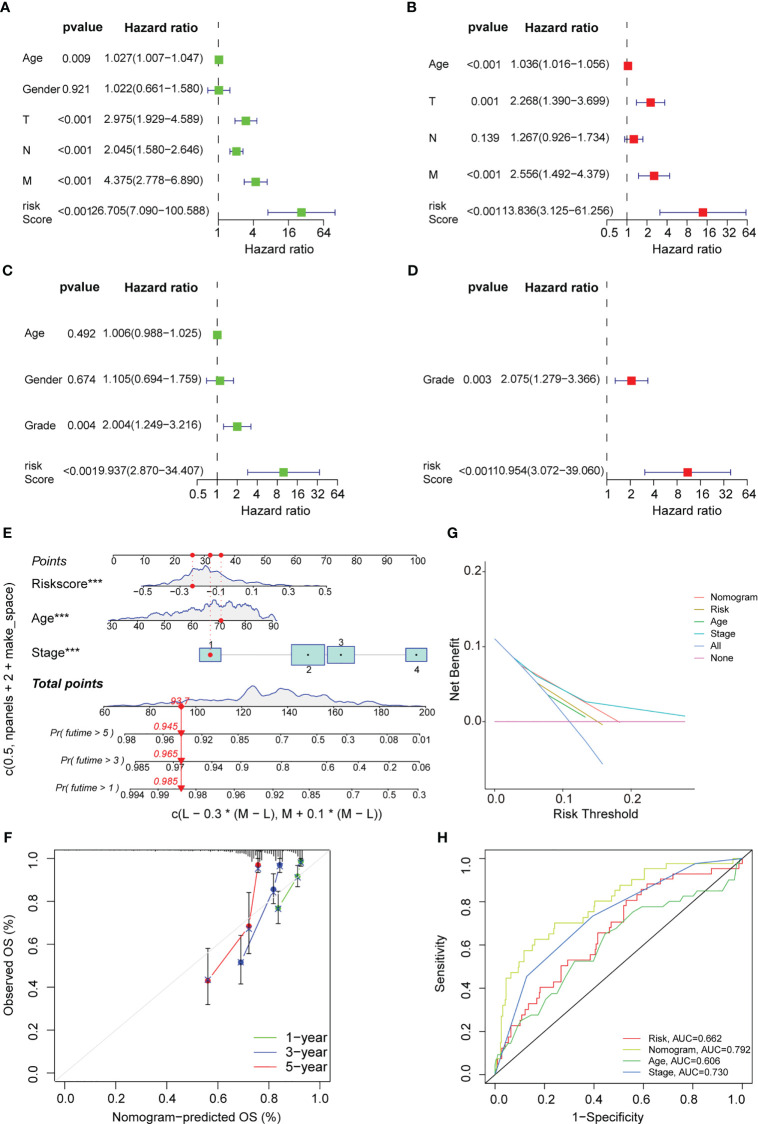
Cox regression analysis and nomogram development. Univariate Cox regression analysis displaying the association between patients’ overall survival and clinicopathological parameters along with the PAEG risk score in training set **(A)** and test set **(C)**. Multivariate Cox regression analysis revealing independent prognostic factors in training set **(B)** and test set **(D)**. **(E)** Nomogram depending on the PAEG risk score and other clinicopathologic feature predicting the 1 -, 3 - and 5-year overall survival for COAD patients. **(F)** Calibration curves uncovering the consistency between predicted and observed 1-, 3- and 5-year overall survival rates in COAD patients based on the nomogram. DCA curve **(G)** and ROC curve **(H)** of nomogram, risk and other clinicopathologic feature in COAD.

In this study, univariate and multivariate Cox regression analyses were carried out to determine if the risk model could serve as an outstanding independent prognostic signature. The findings of the univariate Cox regression analysis implied that some factors like age, T stage, M stage, N stage, and risk score were significantly and positively related to OS in the training set (**Figure 7A**). Furthermore, a strong and positive relationship was observed between the risk score, grade, and OS in the test set (**Figure 7C**). On the other hand, the multivariate regression analysis of the significant factors involved in the univariate analysis indicated that characteristics such as age, M stage, T stage, and risk score in the training set were significantly linked to OS (**Figure 7B**), while risk score and grade in the test set were significantly and positively related to OS (**Figure 7D**). The aforementioned findings demonstrated that the risk prediction model was an effective and independent predictor that outperformed clinical factors such as age and TNM stage.

Thereafter, based on the above findings, a nomogram was developed in this study, which included factors like stage, age, and risk score (**Figure 7E**). Also, calibration curves were plotted for the nomogram, where the results indicated that all three calibration curves (1-, 3-, and 5-year) were close to the standard curve, thus demonstrating an acceptable predictive effect (**Figure 7F**). The DCA curve indicated that the nomogram was much more beneficial than the extreme curves (**Figure 7G**). The ROC curves indicated that AUC values for the nomogram, age, risk, and stage were 0.792, 0.662, 0.606, and 0.730, respectively (**Figure 7H**). The nomogram curve exhibited a greater AUC value compared to the risk score curve, age curve, and stage curve, suggesting that the nomogram was a better predictor of prognosis.

### Immune infiltration analysis, MCP counter, and drug sensitivity of the PAEG risk model

**Figure 8:**
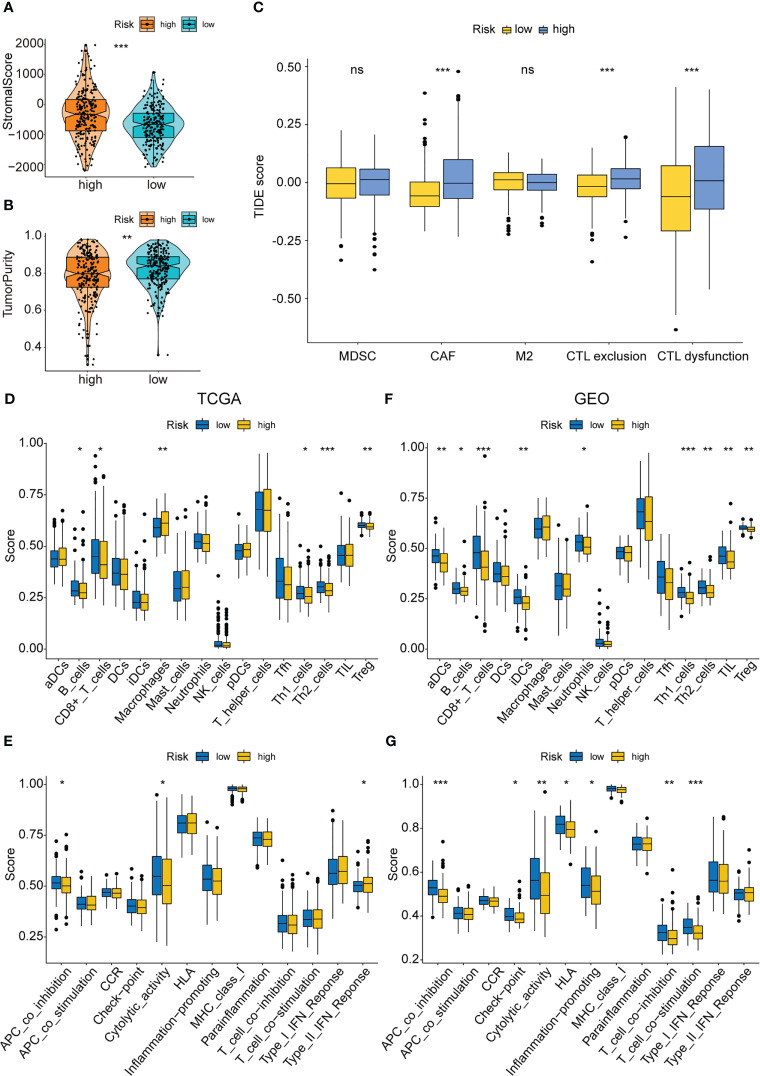
Immune infiltration analysis of the PAEG risk model. **(A, B)** The violin and box plot displaying the difference of the stromal score and tumor purity in two groups. **(C)** TIDE analysis showing the difference of tumor immune dysfunction and exclusion in two groups. The infiltrating levels of 16 immune cell types in training set **(D)** and test set **(F)** estimated by ssGSEA. The infiltrating levels of 13 immune functions in training set **(E)** and test set **(G)** estimated by ssGSEA. ns means p>0.05, *p< 0.05, **p< 0.01, and ***p< 0.001.

**Figure 9:**
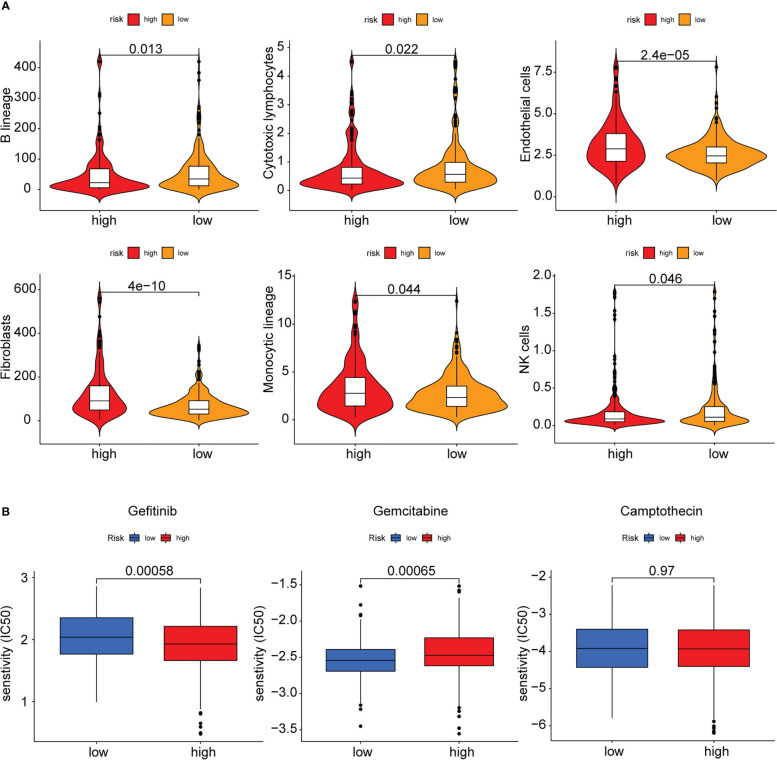
MCPcounter and drug sensitivity analysis of low-risk and high-risk group. **(A)** The violin diagram showing the abundance of 6 types of differentially expressed immune and stromal cells between two groups using MCPcounter. **(B)** Sensitivity difference of three common chemotherapeutic drugs in two groups.

Earlier studies have shown that the TME was linked to COAD distant metastasis, immunotherapy response, and drug sensitivity (22–24). The content of stromal cells present in the TME is indicated by the stromal score. The immune score indicates the content of immune cells in the TME. The high-risk patients showed higher stromal cell levels (**Figure 8A**), however, no remarkable difference was observed in the proportion of immune cells between the low- and high-risk groups (**Supplementary Figure 2A**). A higher tumor purity is linked to a better prognosis as it indicates the proportion of tumor cells present in the tissue. The tumor purity was higher in the low-risk group (**Figure 8B**). TIDE scores were used to assess the potential of tumor immune infiltration in the gene expression profile of the malignant samples and could anticipate the response to the immune checkpoint blockade therapy. The high-risk patients showed enhanced expression of the cytotoxic T lymphocyte (CTL) exclusion, cancer-associated fibroblasts (CAF), and CTL dysfunction (**Figure 8C**). The ssGSEA technique was then used to investigate the infiltration status of 16 immune cells and derive the scores of 13 immunological functions to further assess the relationship between immune infiltration and the risk model. The low-risk patients displayed a higher infiltration of CD8^+^ T cells, Th1 cells, B cells, Th2 cells, and Treg cells as well as higher immunological functions like APC_co_inhibition and Cytolytic_activity. On the other hand, the high-risk patients displayed a higher infiltration level of macrophages and immunological functions like Type_II_IFN_response in the training set (**Figures 8D, E**). In the test set, the low-risk group displayed higher immunological functions such as Check-point, Cytolytic_activity, APC_co_inhibition, HLA, T_cell_co_inhibition, Inflammation-promoting, and T_cell_co_stimulation. They also showed a higher infiltration level of aDCs, B cells, Neutrophils, TILs, iDCs, Th2 cells, Th1 cells, CD8^+^ T cells, and Treg cells (**Figure 8F, G**). Then, the MCPcounter software was used for comparing the contents of 10 immune and stromal cells in the two groups. The high-risk group contained a higher number of endothelial cells and fibroblasts, whereas the low-risk group contained more B lineage, cytotoxic lymphocytes, and NK cells (**Figure 9A**, **Supplementary Figure 2B**). Also, 10 common immune checkpoint molecules and 24 major histocompatibility complex (MHC) molecules in the high- and low-risk groups were evaluated, and no remarkable differences were noted (**Supplementary Figure 2C, D**).

Finally, the sensitivity of the high- and low-risk patients to the common therapeutic drugs used in COAD was evaluated, and the findings implied that the high-risk patients showed a higher sensitivity to gemcitabine, while the low-risk patients showed a higher sensitivity to gefitinib. Both groups showed no difference in their sensitivity to camptothecin (**Figure 9B**).

### Validation of PAEG in the risk model in cell and tissue experiments

**Figure 10:**
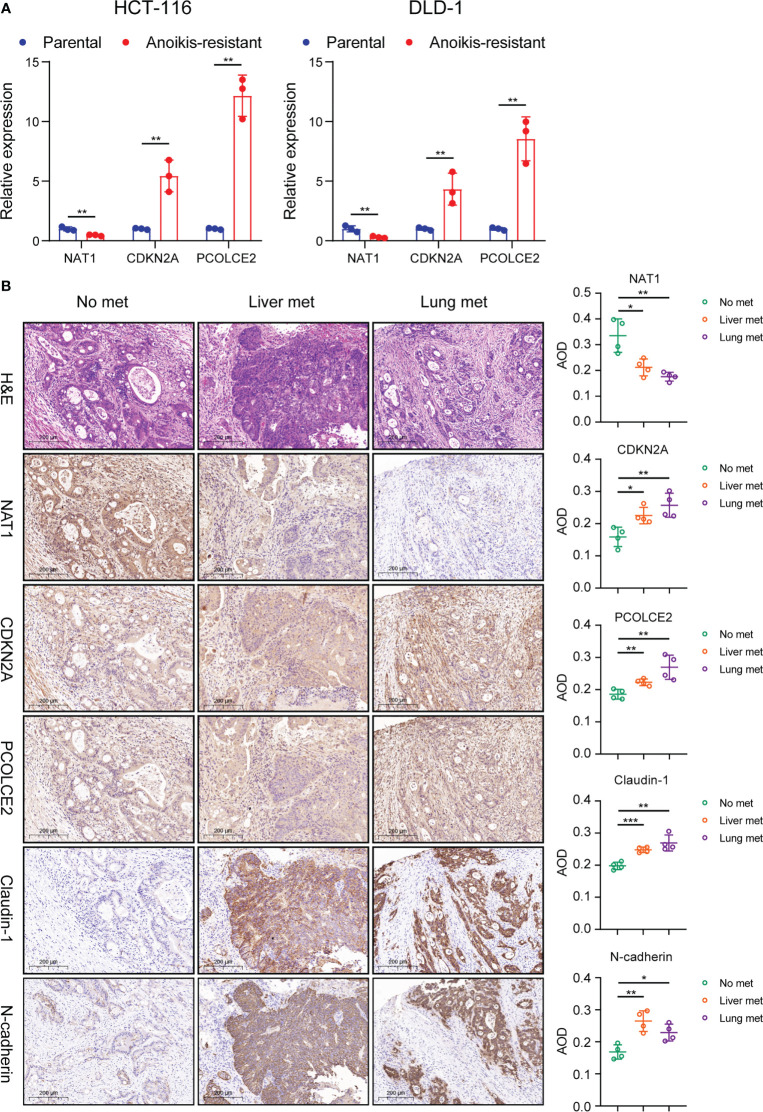
Validation of PAEG in the risk model in cell and tissue experiments. **(A)** Relative expression of NAT1, CDKN2A and PCOLCE2 in the parental and anoikis-resistant groups by qPCR. **(B)** The protein expression of NAT1, CDKN2A, PCOLCE2, Claudin-1 and N-cadherin in the primary tumor in the no distant metastasis group (n=4), liver metastasis group (n=4) and lung metastasis group (n=4) were detected by IHC (scale bars, 200 μm.) and the AOD values were expressed as scatter plots. *p< 0.05, **p< 0.01, and ***p< 0.001.

In this study, the anoikis-resistant COAD cell lines such as HCT-116 and DLD-1 were constructed. Then, the mRNA expression levels of NAT1, CDKN2A, and PCOLCE2 in the parental and anoikis-resistant groups were detected separately by means of qPCR. The findings of this experiment indicated that the NAT1 mRNA expression level was decreased, whereas the CDKN2A and PCOLCE2 mRNA expression levels were elevated in the anoikis-resistant group, which led to the conclusion that NAT1 could facilitate anoikis, while CDKN2A and PCOLCE2 were responsible for anoikis resistance (**Figure 10A**). Considering the significant role of PAEGs in the distant metastasis of COAD, four cases of primary COAD tissues without distant metastasis, with liver metastasis, and with lung metastasis were selected, respectively, to detect the protein expression levels of the above-mentioned three genes by IHC. An earlier study showed that Claudin-1 could attenuate E-cadherin expression in colorectal cancer by upregulating ZEB-1, which, in turn, promoted EMT and reduced anoikis (13). Also N-cadherin is one of the key markers of EMT. Hence, Claudin-1 and N-cadherin were used as references and compared to the three PAEGs. The results implied that the NAT1 protein expression level was elevated in the tissues without distant metastasis compared to the tissues with liver and lung metastasis, whereas the CDKN2A and PCOLCE2 protein expression levels were increased in the tissues with liver and lung metastasis. The above findings indicated a positive correlation between high anoikis resistance, high EMT, and more distant metastasis of COAD (**Figure 10B**).

## Discussion

Distant metastases severely limit the prognosis of COAD patients. Although current therapeutic approaches are more mature, a recent meta-analysis suggested that median recurrence-free survival was 1.3 years after resection of colorectal liver metastases (25). A high recurrence rate is an important cause of death for COAD patients. Most patients with distant metastases are not detected during the initial diagnosis and suffer a worse prognosis. Therefore, it is essential to investigate techniques that help in preventing distant metastasis of COAD. According to existing research (26, 27), the first step of distant metastases of COAD involves the invasion of tumor cells into the stromal environment. Both anoikis and EMT play important roles in inhibiting and promoting invasion during this phase; therefore, an in-depth study of the crosstalk between anoikis and EMT can help uncover key genes involved in distant metastasis.

In this study, API and EPI were quantified for determining the anoikis and EMT levels in COAD, using the PCA technique, and the group that showed the worst survival curve (i.e., the API Low + EPI High group) was isolated. This was in agreement with an earlier finding that anoikis repressed metastasis and EMT promoted metastasis (28, 29). This is the first study that used this technique for clustering COAD patients, and a favorable prognostic difference presents the rationality and effectiveness of this type of grouping technique, which needs to be further analyzed and experimentally validated.

Next, a risk model was developed using three genes: NAT1, CDKN2A, and PCOLCE2. Arylamine N-acetyltransferase 1 (NAT1) was shown to catalyze the N- or O-acetylation of different arylamine and heterocyclic amine substrates and was observed to be involved in tumor progression and chemotherapy resistance (30). MicroRNA-6744-5p facilitated anoikis and targeted NAT1 in breast cancer cells (31). NAT1 expression levels also correlated with EMT status and metastatic behavior in breast cancer patients (32). Moreover, by interacting significantly with CDK4 and CDK6, the cyclin-dependent kinase inhibitor 2A (CDKN2A) functioned as a negative regulator of the proliferation of normal cells (33). CDKN2A also played a vital role in regulating the anoikis in hepatocellular carcinoma cells (34) and pancreatic cancer cells (35–38) and hence could serve as an anoikis-related signature gene to predict the prognosis of endometrial carcinoma patients (39). Meanwhile, CDKN2A may facilitate colorectal cancer cell metastasis through the induction of EMT and may be associated with the infiltration status of multiple immune cells (40, 41). Procollagen C-endopeptidase enhancer 2 (PCOLCE2) refers to a collagen-binding protein that binds to the C-terminal pro-peptide of types I and II procollagens and could enhance the cleavage of the pro-peptide by BMP1 (42). PCOLCE2 can be considered as an EMT-linked gene for anticipating the prognosis of gastric cancer patients (43, 44) and the metastasis ability of COAD (45). However, none of the studies determined the role of PCOLCE2 in anoikis. The risk model was used for categorizing the COAD patients into the high- and low-risk categories, and the OS duration of the patients with different clinicopathological parameters like gender, age, and stage was seen to be significantly different between both categories. The nomogram curve that was developed using the risk model was more sensitive and specific in reflecting the prognosis of COAD patients than age, stage, and risk model.

Furthermore, the stromal and immune infiltration analysis was carried out based on the risk model. The findings revealed that this risk model was associated with a few acquired immune cells like Th1 cells, Th2 cells, B cells, and Treg cells, and with the innate immune cells like lymphocytes, monocytes, and NK cells, but not with numerous MHC molecules and immune checkpoint genes. However, it showed a stronger correlation with stromal infiltrates such as CAF and endothelial cells. Anoikis resistance and EMT are related to cell adhesion and ECM remodeling, while stromal cells are involved in the composition and remodeling of ECM (46–49). Hence, the risk model can better reflect the differences in stromal cell distribution, and the specific molecular mechanisms deserve further investigation.

Lastly, our cell experiments confirmed the relationship between the expression of NAT1, CDKN2A, PCOLCE2, and anoikis, suggesting that NAT1 promoted anoikis while CDKN2A and PCOLCE2 contributed to anoikis resistance in COAD cells. IHC was used for verifying the link between these PAEGs and distant metastasis of COAD, and the results also suggested that the high NAT1 expression level was associated with no distant metastasis while the high CDKN2A or PCOLCE2 expression levels were related to hepatopulmonary metastasis. The above findings were in agreement with the findings of the univariate Cox analysis of these genes in TCGA.

This study has some limitations despite its superiorities. Firstly, we obtained the EMT signature containing 198 genes from the MSigDB portal in where some important genes of EMT such as ZEB1, ZEB2, SNAI1 are not included. This may bring some uncertainties, and a more complete database may yield better analysis results. Secondly, the ROC curves for the 1-year, 3-year, and 5-year risk scores were<0.7 in the training and test sets, indicating that the prediction rate was less accurate, and the risk model needs further improvement. Thirdly, after classifying the patients into the API Low + EPI High and the others group, the immune infiltration, stromal infiltration, and drug sensitivity rates in the two groups were determined by means of gene enrichment and DEG analyses. The ESTIMATE tool indicated no significant difference in the immune cell proportion between the low- and high-risk groups, it should be noted that the analysis did not include some important immune cells such as dendritic cells or NK cells. Fourthly, the study found no significant difference in the expression of 10 immune checkpoint molecules and 24 MHC molecules between the low- and high-risk groups, suggesting that the immune checkpoint blockade therapy response may not be affected by the risk model. Finally, the molecular mechanisms of the three PAEGs in anoikis and EMT need to be explored and validated by further *in vivo* and *in vitro* experiments. Additionally, the univariate Cox analysis of PCOLCE2 revealed that it is a poor predictive gene, although its expression is low in tumor tissues relative to normal tissue, which is an intriguing and paradoxical finding. More research is needed to determine its role and mechanism in colorectal cancer, as very few studies have investigated the role played by this gene. Hence, more research needs to be conducted to determine its role and mechanism in the future.

This is the first study that combines the anoikis and EMT analysis based on distant metastasis of COAD. Herein, a favorable molecular group and prognostic risk model was constructed, which can help to establish biomarkers and drug intervention targets for patients with distant metastasis. Moreover, drug sensitivity analyses were conducted in this study, which can provide some clinical drug references for these patients.

## Conclusions

Collectively, based on the anoikis and EMT gene sets, the prognosis-related ARGs and ERGs were identified using univariate Cox analysis, and then PCA stratified analysis was used for categorizing the patients into two groups based on the greatest prognostic differences. A prognostic risk model was constructed, which showed good predictive sensitivity and specificity. This risk model was linked to the tumor microenvironment, especially stromal infiltration. Finally, the findings in this study could help in developing novel molecular biomarkers, therapeutic targets, and clinical medicines for treating patients with COAD metastasis.

## Data availability statement

The original contributions presented in the study are included in the article/**Supplementary Material**. Further inquiries can be directed to the corresponding authors.

## Ethics statement

The studies involving human participants were reviewed and approved by the Ethical Committee of The Affiliated Suzhou Hospital of Nanjing Medical University. The patients/participants provided their written informed consent to participate in this study.

## Author contributions

JZhou, SYang, DZhu and HLi for the acquisition of data, analysis and interpretation of data, statistical analysis and drafting of the manuscript. XMiao, MGu, WXu, YZhang, WTang, JZha, RShen and JZhu for technical and material support. JZhou, ZYuan, and XGu for study concept and design, obtained funding and study supervision. All authors read and approved the final manuscript.
