# Supplementary material for: The crosstalk between anoikis and epithelial-mesenchymal transition and their synergistic roles in predicting prognosis in colon adenocarcinoma
Source: Front Oncol. 2023 Jun 7;13:1184215. doi: 10.3389/fonc.2023.1184215 (PMC10284081; doi:10.3389/fonc.2023.1184215)
Supplement: Supplementary file 1 [file DataSheet_1.docx]

**Supplementary Figure**

**
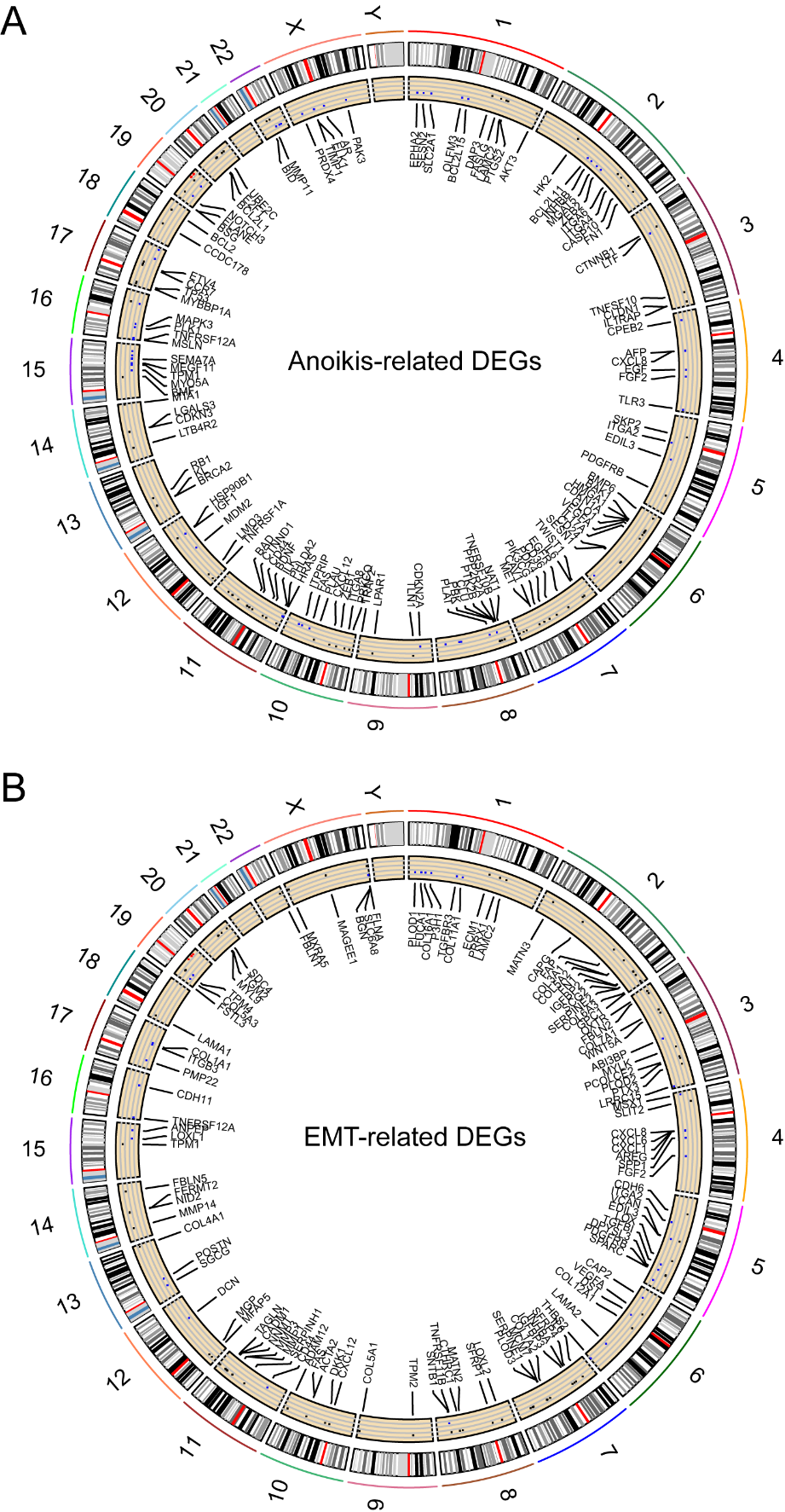
**

**Supplementary Figure 1 |** The locations of diff-ARGs (A) and diff-ERGs (B) on human chromosomes.

**
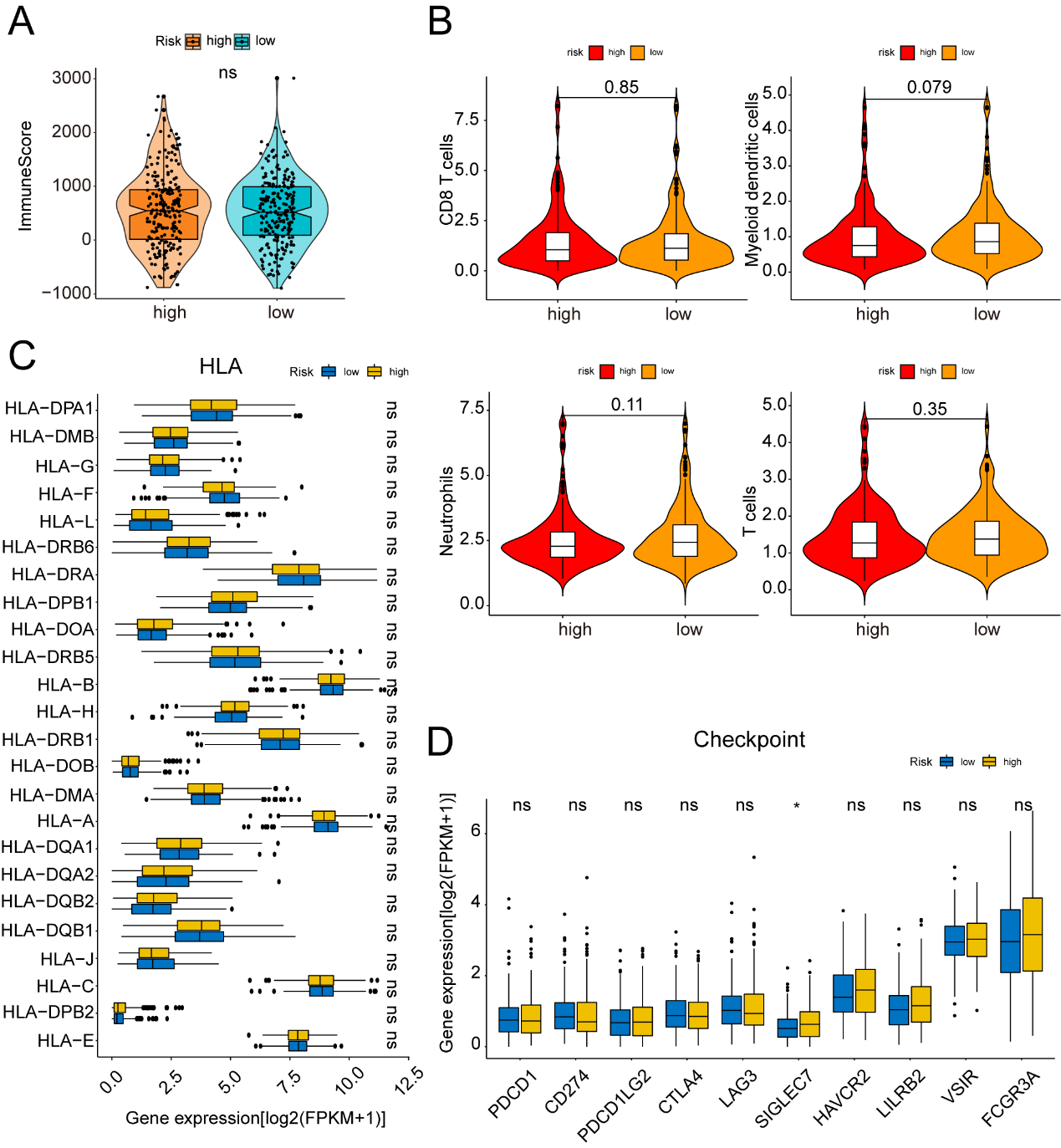
**

**Supplementary Figure 2 |** (A) The violin and box plot displaying the difference of the immune score in two groups. (B) The violin diagram showing the abundance of 4 types of no differentially expressed immune and stromal cells between two groups using MCPcounter. The gene expression of 24 MHC molecules in train set(C) estimated by HLA. The gene expression of 10 common immune checkpoint molecules in train set(D) estimated by Checkpoint. ns means p>0.05 and *p < 0.05.
