# Supplementary material for: The crosstalk between anoikis and epithelial-mesenchymal transition and their synergistic roles in predicting prognosis in colon adenocarcinoma
Source: Front Oncol. 2023 Jun 7;13:1184215. doi: 10.3389/fonc.2023.1184215 (PMC10284081; doi:10.3389/fonc.2023.1184215)
Supplement: Supplementary file 2 [file Table_1.docx]

Table S1: the primer sequences used for qRT-PCR

| Primer | Forward (5’-3’) | Reverse (5’-3’) |
| --- | --- | --- |
| NAT1 | GCGGCAGCCATATGGACATTGAAGCATA | TCGAGTGCGGCCGCCTAAATAGTAAAAAATCTATCACC |
| CDKN2A | CTTCCTCGGGTGCC | ACCCCTTCATTGCTAC |
| PCOLCE2 | GCAGTGAAGGTTTTCCTGGAGTG | AGTCATAGCGGCACAGGTTGTC |
| GAPDH | ACACCCACTCCTCCACCTTT | TTACTCCTTGGAGGCCATGT |
