# Supplementary material for: The crosstalk between anoikis and epithelial-mesenchymal transition and their synergistic roles in predicting prognosis in colon adenocarcinoma
Source: Front Oncol. 2023 Jun 7;13:1184215. doi: 10.3389/fonc.2023.1184215 (PMC10284081; doi:10.3389/fonc.2023.1184215)
Supplement: Supplementary file 3 [file Table_2.docx]

Table S2: the antibodies used for IHC

| Antibodies | Item number | Company | Host | Dilution |
| --- | --- | --- | --- | --- |
| NAT1 | 19188-1-AP | Proteintech | Rabbit | 1:100 |
| CDKN2A | 10883-1-AP | Proteintech | Rabbit | 1:1000 |
| PCOLEC2 | HPA013203 | Sigma-Aldrich | Rabbit | 1:100 |
| Claudin-1 | #13255T | CST | Rabbit | 1:400 |
| N-cadherin | #13116T | CST | Rabbit | 1:200 |
